# A Case Report of Pneumoretroperitoneum from Blunt Trauma in a Patient with Chronic Obstructive Pulmonary Disease

**DOI:** 10.5811/cpcem.1239

**Published:** 2023-10-18

**Authors:** Annemarie Daecher, Brittany Hartman, James Krueger

**Affiliations:** * Albert Einstein Medical Center, Department of Emergency Medicine, Philadelphia, Pennsylvania; † Albert Einstein Medical Center, Department of Toxicology, Philadelphia, Pennsylvania

**Keywords:** *case report*, *pneumoretroperitoneum*, *pneumomediastinum*, *blunt trauma*

## Abstract

**Introduction:**

Pneumomediastinum is a rare complication of blunt traumatic injury and is thought to be due to the Macklin effect, a pathophysiologic process comprised of three steps: alveolar rupture secondary to blunt injury; air dissecting along bronchovascular sheaths; and spread of pulmonary interstitial edema into the mediastinal space. Pneumomediastinum is rarely associated with pneumoretroperitoneum.

**Case Report:**

We present a case of a patient who suffered a cardiac arrest after a fall during a chronic obstructive pulmonary disease exacerbation, leading to pneumoretroperitoneum.

**Conclusion:**

This case highlights the complications that can arise from blunt trauma and how underlying lung pathology can worsen these complications.

CPC-EM Capsule What do we already know about this clinical entity?

*Pneumoretroperitoneum is a rare diagnosis that represents a barrier breakdown between the retroperitoneum and an air-containing space, typically due to disease.*

What makes this presentation of disease reportable?

*There are very few reports of pneumoretroperitoneum occurring with blunt traumatic injury, and we present a novel combination of cardiac arrest, chronic obstructive pulmonary disease (COPD), and trauma.*

What is the major learning point?

*Underlying lung pathology, such as COPD, can contribute to worse outcomes in traumatic injury, and we highlight one such complication that can arise in this setting.*

How might this improve emergency medicine practice?

*This case highlighting a complication from blunt traumatic injury may help others quickly identify and address this complication.*

## INTRODUCTION

Pneumomediastinum is a relatively common injury found after blunt thoracic trauma and portends a higher degree of severe injury with a higher complication risk than those who do not develop pneumomediastinum following blunt thoracic injury.^1^ Pneumomediastinum was proposed by Macklin to be caused through a stepwise downstream effect of a sudden increase in intrathoracic pressure that causes alveolar rupture and subsequent emphysematous dissection of air along the bronchovascular sheaths, which spreads into the mediastinum. The first symptom of pneumomediastinum is most commonly chest pain, followed closely by dyspnea as the second most common presenting symptom. It is more common in males and more commonly associated with underlying lung disease such as asthma.^2^ After development of pneumomediastinum, air can dissect into the mediastinum along the parietal pleura, leading to pneumothorax. Air can further dissect along the great vessels or the esophagus through the diaphragmatic hiatus and cause pneumoretroperitoneum.^3^

Pneumoretroperitoneum is most often described in the literature as being associated with respiratory tract rupture or rupture of alveoli, infection with gas-forming organisms, or interruption of the barriers between the gastrointestinal tract and the retroperitoneal space, commonly after colonoscopy or surgical manipulation of the gastrointestinal tract.^4^ Pneumoretroperitoneum is rarely associated with blunt traumatic injury, such that in one study, only one patient in a cohort of 233 patients with blunt abdominal trauma was noted to have pneumoretroperitoneum.^5^ Although it appears that both pneumomediastinum and pneumoretroperitoneum have been found in patients with blunt traumatic injury, they only rarely occur together, and barotrauma from ventilation can subsequently cause extension of underlying injury, resulting in high peak pressures and significant difficulty in adequate ventilation of these patients.^6^

## CASE REPORT

A 50-year-old female patient presented to the emergency department (ED) in significant respiratory distress. She had a known past medical history of tobacco dependence, chronic obstructive pulmonary disease (COPD), gastroesophageal reflux disease, and hypertension. On arrival, the patient was noted to be apneic and was actively being ventilated with a bag-valve-mask ventilation. She rapidly progressed from an irregularly irregular cardiac rhythm to sinus bradycardia, and then into a pulseless electrical activity (PEA) arrest within minutes of arrival. She underwent approximately 30 minutes of cardiopulmonary resuscitation, with endotracheal intubation performed via video laryngoscopy. She was noted to have significant resistance to bagging after intubation, and after return of spontaneous circulation, was noted to have high peak pressures on the ventilator and was persistently hypotensive.

On examination, the patient was noted to have distant, rhonchorous, and wheezy breath sounds bilaterally, and she was treated for her bronchospasm with nebulized albuterol-ipratropium solution, continuous nebulized albuterol, magnesium sulfate, methylprednisolone, subcutaneous terbutaline, and titratable epinephrine (to also assist with her hypotension). She was noted to have continuously high peak pressures; so, an intravenous push of vecuronium was given without improvement in ventilation and with continuously high peak airway pressures noted on the ventilator. She had an electrocardiogram concerning for new-onset atrial fibrillation with rapid ventricular response, left axis deviation, a new right bundle branch block, and a left anterior fascicular block with signs of right heart strain. Given her difficulty with ventilation with high peak pressures and hypotension, she was taken for a computed tomography (CT) with pulmonary embolism protocol as there was concern that the etiology of her arrest may have been a massive pulmonary embolism.

**Image 1.**
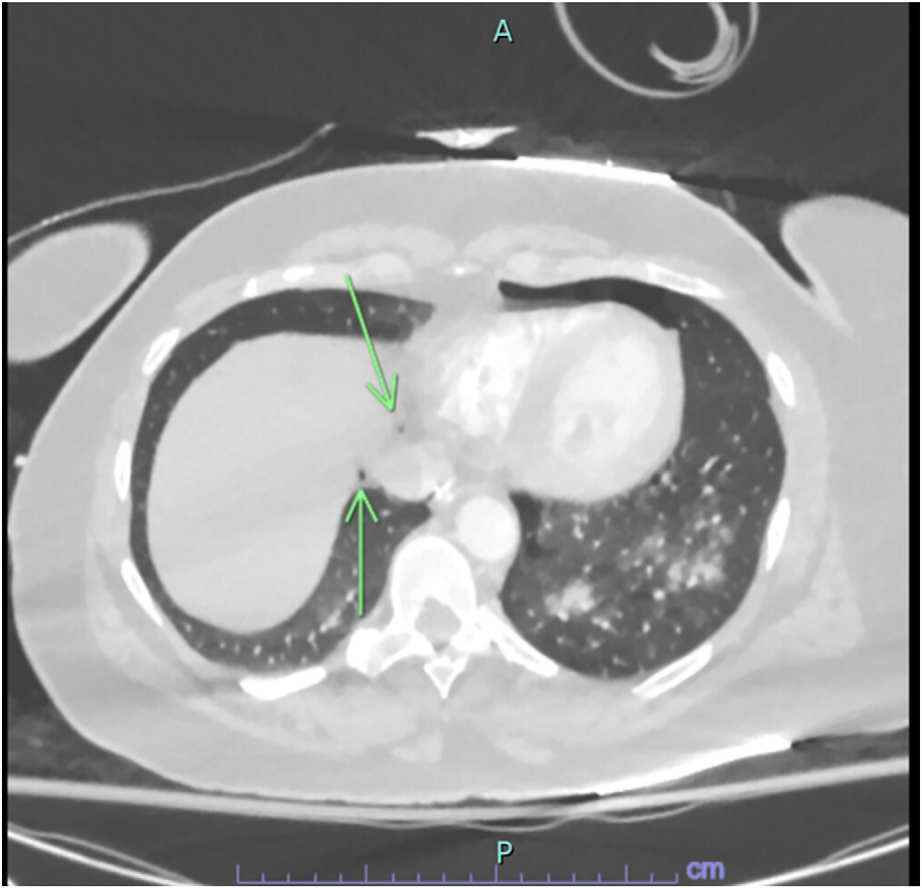
Initial computed tomography with pulmonary embolism protocol performed, revealing pneumomediastinum in the lower portion of the mediastinum (green arrows) favored to be secondary to alveolar rupture as well as a left-sided pneumothorax.

**Image 2.**
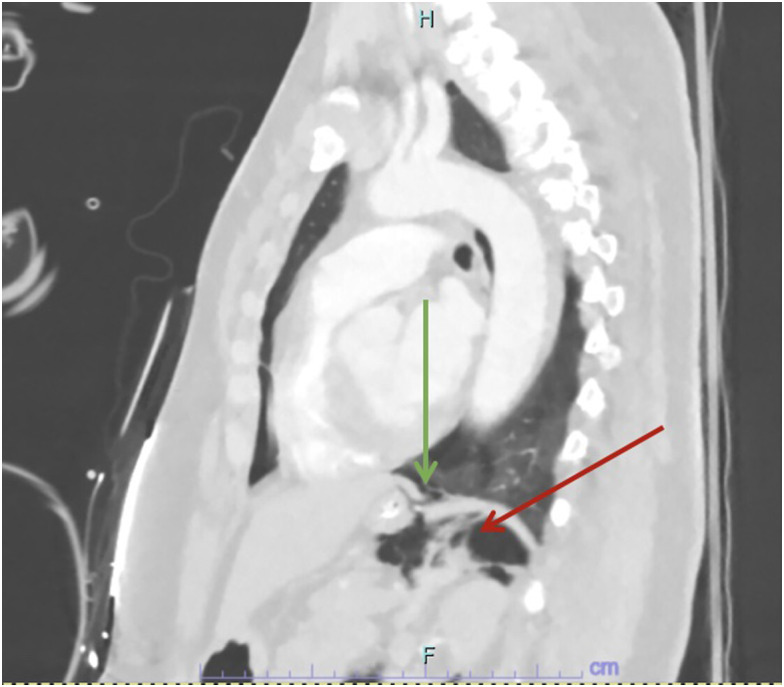
The initial computed tomography with pulmonary embolism protocol performed on the day of presentation demonstrated discontinuity of the medial left hemidiaphragm **posteriorly** (green arrow) with air tracking from the lower mediastinum into the left retroperitoneal space posterior to the stomach (red arrow).

**Image 3.**
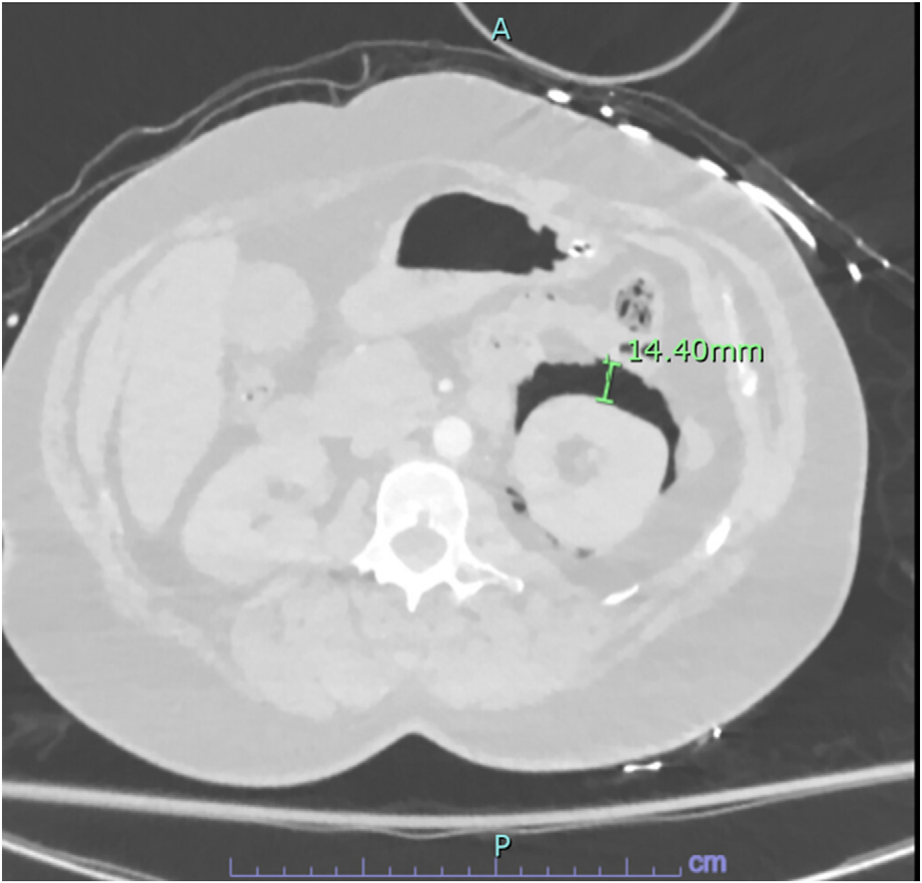
Image from patient’s computed tomography (CT) with pulmonary embolism protocol performed on the day of presentation revealing air in the left perinephric space, which is likely related to retroperitoneal extension of air from the patient’s pneumomediastinum as a CT of the abdomen and pelvis was performed and revealed no other acute traumatic injuries in the abdomen or pelvis that could account for the free air.

This revealed no evidence of pulmonary embolism; however, it did show evidence of alveolar rupture with pneumomediastinum consistent with the Macklin effect and a small left-sided pneumothorax (Image 1) with associated medial left hemi diaphragmatic rupture (Image 2) with pneumoretroperitoneum tracking along the left upper abdomen and left perinephric space with left-sided nondisplaced rib fractures of the fifth and sixth ribs (Image 3). A CT of the abdomen and pelvis was then performed, which showed a small amount of free air consistent with pneumoretroperitoneum adjacent to the gastric cardia, left kidney, and left adrenal gland, but with no definitively identified intra-abdominal traumatic injury.

Further history from the family, who arrived several hours after the patient’s initial presentation, revealed she had been complaining of difficulty breathing earlier in the day, and she subsequently suffered a witnessed fall down a flight of stairs with head trauma and apparent loss of consciousness. The patient’s physical examination revealed no external signs of trauma on initial arrival.

The patient was admitted to the medical intensive care unit in the setting of cardiac arrest with prolonged down time, with improvement in her pneumomediastinum and pneumoretroperitoneum with lung-protective ventilation strategies and paralytics. The patient’s treatment for her COPD exacerbation was extensive and included continuous albuterol nebulization of 40 milligrams (mg) over four hours, intermittent scheduled three-milliliter ipratroprium-albuterol nebulizers every six hours, 0.25 mg of budesonide twice daily, 0.5 mg of ipratroprium four times daily, isolated two mg of magnesium sulfate administration daily if worsening wheeze on examination, 80 mg of methylprednisolone daily, a one-time dose of 0.25 mg of subcutaneous terbutaline, and a one-time dose of one microgram (μg) of epinephrine. Sedation was maintained with both ketamine at 1.5 mg per kilogram (kg) per hour and propofol at 20 μg/kg per minute. For the patient’s hypotension, she was treated with epinephrine as the first-line vasopressor choice given its underlying beta adrenergic effects in the setting of her profound bronchospasm.

However, the patient’s cardiac arrest resulted in severe hypoxic brain injury, leading to subsequent diffuse cerebral edema with effacement of the basal cisterns and tonsillar herniation seen on CT of her head. She became increasingly hypertensive and was weaned off vasopressors and started on titratable nicardipine at a maximum of 12.5 mg per hour and was given a hypertonic saline bolus. The patient lost all evidence of brainstem reflexes five days after suffering cardiac arrest but continued to trigger some spontaneous breaths on the ventilator. Multiple family meetings were held regarding patient prognosis, and ultimately the patient was palliatively extubated with subsequent demise 26 days after arrival.

## DISCUSSION

Our patient presented to the ED unresponsive and in severe respiratory distress and shortly afterwards went into PEA cardiac arrest which was followed by multiple rounds of cardiopulmonary resuscitation. After intubation, the patient was found to have significant resistance to bagging and significantly elevated peak pressures, which was initially thought to be secondary to positive end-expiratory pressure caused by the progressive accumulation of air, breath stacking, and the patient’s underlying COPD. A CT with pulmonary embolism protocol was performed, which revealed non-displaced rib fractures along the anterior aspects of the left fifth and sixth ribs with a small, left-sided pneumothorax anteriorly. There was also discontinuity of the medial left hemidiaphragm posteriorly with air tracking from the lower mediastinum into the left retroperitoneal space from the lower mediastinum. While it is unclear whether the patient suffered her rib fractures (and subsequently left anterior pneumothorax) from cardiopulmonary resuscitation or from her fall, the instability of the patient at presentation suggests she suffered these rib fractures prehospital.

The exact etiology of the patient’s diaphragmatic rupture and pneumoretroperitoneum is also unclear but could have been directly caused by blunt trauma, iatrogenically during resuscitation, or secondarily caused through extension of her pneumomediastinum, which likely was secondary to the Macklin effect and was further exacerbated by positive pressure ventilation. The fact that the patient’s rib fractures were located anteriorly and her diaphragmatic rupture was posterior in nature also suggests the diaphragmatic rupture was not necessarily directly caused by her traumatic injuries. In review of the literature, pneumoretroperitoneum appears to be a rare diagnosis, especially in the setting of blunt trauma with no identifiable abdominal injuries.

Diaphragmatic injury also appears to be somewhat rare in the setting of blunt trauma. In fact, a study performed by Fair et al, published in the *American Journal of Surgery*, analyzed the incidence of diaphragmatic injury secondary to trauma and found that of 3,783 patients diagnosed with a traumatic diaphragmatic injury, only 33% of these patients suffered a diaphragmatic injury secondary to blunt trauma with only 7.6% of these patients suffering diaphragmatic injury secondary to falls.^7^ Mortality was also significantly higher in patients with blunt traumatic diaphragmatic injury (19.8%) compared to patients with traumatic diaphragmatic injury secondary to penetrating trauma (8.8%), demonstrating the importance of rapid identification of diaphragmatic injury due to blunt trauma. In patients without a history of trauma, pneumoretroperitoneum is usually secondary to duodenal ulcer perforation, colonic perforation, retrocecal appendicitis, emphysematous cholecystitis, or secondary to iatrogenic causes of duodenal or colonic perforation due to endoscopic retrograde cholangiopancreatography and colonoscopy, respectively.^8^

Pneumoretroperitoneum secondary to pneumomediastinum is also a rare complication of status asthmaticus. While the incidence of pneumomediastinum secondary to asthma exacerbations is unknown, a small study done by Vianello et al revealed that five of 45 patients diagnosed with a severe asthma exacerbation also were diagnosed with pneumomediastinum.^9^ None of the patients with pneumomediastinum required needle decompression, surgical intervention, or chest tube placement, and there were no in-hospital deaths; they had similar length of hospital stays as their counterparts without pneumomediastinum. Other studies have found the incidence to be much lower (one in 30,000).^10^ The main treatment strategy employed for the patient’s pneumomediastinum, pneumoretroperitoneum, and hypercapnic respiratory failure was lung protective ventilation. The patient’s ventilator settings were continuously readjusted based on arterial blood gas findings that were performed daily and in response to changes in clinical status. The patient was also intermittently maintained on cisatracurium 0.1 mg/kg per hour to help maintain synchronicity with the vent, as occasionally the patient would hyperventilate resulting in breath stacking, which worsened her underlying respiratory failure. The patient had interval improvement in her pneumoretroperitoneum, pneumomediastinum, and pneumothorax using this closely monitored ventilatory strategy in conjunction with pulmonary critical care-trained intensivists.

When the patient’s CT findings revealed evidence of tonsillar herniation, given worsening of her anoxic brain injury, her family elected for palliative extubation, and the patient passed peacefully after palliative extubation with family at bedside and discontinuation of all sedating medications in line with institutional protocols.

## CONCLUSION

Whether traumatic or spontaneous in nature secondary to our patient’s underlying history of obstructive lung disease, her pneumoretroperitoneum was thought to be secondary to the Macklin effect (causing pneumomediastinum) as well as via the defect in the left hemidiaphragm, given the patient had no abdominal or pelvic traumatic injuries identified. It is entirely possible that the patient’s pneumoretroperitoneum was in fact secondary to profound intrathoracic pressures secondary to her COPD, alveolar rupture, pneumothorax, and positive pressure ventilation, which through the Macklin effect caused pneumomediastinum and subsequent pneumoretroperitoneum. The critically ill nature of the patient on presentation to the ED stresses the importance of prompt identification of this pathology to help guide management.
